# Combined congenic mapping and nuclease-based gene targeting for studying allele-specific effects of *Tnfrsf9* within the *Idd9.3* autoimmune diabetes locus

**DOI:** 10.1038/s41598-019-40898-8

**Published:** 2019-03-13

**Authors:** Matthew H. Forsberg, Bardees Foda, David V. Serreze, Yi-Guang Chen

**Affiliations:** 1 Department of Microbiology and Immunology, Medical College of Wisconsin, Milwaukee, WI 53226 USA; 2 Department of Pediatrics, Medical College of Wisconsin, Milwaukee, WI 53226 USA; 3 Max McGee National Research Center for Juvenile Diabetes, Medical College of Wisconsin, Milwaukee, WI 53226 USA; 4 The Jackson Laboratory, Bar Harbor, Maine, 04609 USA; 5 Department of Molecular Genetics and Enzymology, National Research Centre, Dokki, Egypt; 6 Present Address: Department of Pediatrics, University of Wisconsin School of Medicine and Public Health, Madison, WI 53705 USA

## Abstract

Rodent complex trait genetic studies involving a cross between two inbred strains are usually followed by congenic mapping to refine the loci responsible for the phenotype. However, progressing from a chromosomal region to the actual causal gene remains challenging because multiple polymorphic genes are often closely linked. The goal of this study was to develop a strategy that allows candidate gene testing by allele-specific expression without prior knowledge of the credible causal variant. *Tnfrsf9* (encoding CD137) is a candidate gene for the *Idd9.3* type 1 diabetes (T1D) susceptibility locus in the nonobese diabetic (NOD) mouse model. A C57BL/10Sn (B10)-derived diabetes resistance *Idd9.3* congenic region has been shown to enhance accumulation of CD137^+^ regulatory T cells and serum soluble CD137 in NOD mice. By combining the power of congenic mapping and nuclease-based gene targeting, we established a system where a pair of F1 hybrids expressed either the B10 or NOD *Tnfrsf9* allele mimicking coisogenic strains. Using this approach, we demonstrated that the allelic difference in B10 and NOD *Tnfrsf9* alone was sufficient to cause differential accumulation of CD137^+^ regulatory T cells and serum soluble CD137 levels. This strategy can be broadly applied to other rodent genetic mapping studies.

## Introduction

Rodent models have been widely used to study the genetics of human complex diseases^1,2^. Except for more complicated breeding methods, experimental strategies typically involve a cross between two inbred strains that are respectively resistant and susceptible to a trait to map genetic loci regulating the phenotype. Initial localization of the chromosomal regions is then followed by generation of congenic strains to physically map the underlying genes. While these approaches are effective to define a relatively small region harboring phenotype-modulating genetic variants, identification of the causal genes remains difficult. When several genes are closely linked within a small genetic region, it is not practical to further test them by a traditional backcross breeding scheme due to limited recombination. The underlying gene is often inferred by its expression level and function or the presence of deleterious polymorphisms. The causal variant could be conclusively determined if coisogenic strains respectively express only one of the two parental alleles of a candidate gene. However, this is practically challenging because multiple polymorphisms often exist in the candidate gene and precise genetic manipulation requires prior knowledge of the credible variant.

Nonobese diabetic (NOD) mice develop spontaneous type 1 diabetes (T1D) mimicking the human disease and have been an effective model for identifying pathogenic genetic determinants^3,4^. By outcrossing to other strains, more than 30 autoimmune T1D susceptibility (*Idd*) loci have been identified in NOD mice^3^. Among those, the *Idd9.3* locus has been mapped to the distal end of chromosome 4 containing the CD137-encoding *Tnfrsf9* gene^5,6^. Interestingly, NOD-derived CD137 is hypofunctional compared to the C57BL/10Sn (B10) counterpart, and three nonsynonymous polymorphisms are present between NOD and B10 *Tnfrsf9* alleles^5,6^. NOD mice congenic for the B10-derived *Idd9*.*3* region (NOD.*Idd9*.*3*^*B10*^) are more resistant to T1D^7^. Compared to NOD mice, the NOD.*Idd9*.*3*^*B10*^ congenic strain has significantly higher levels of CD137^+^ FOXP3^+^ regulatory CD4 T cells (Tregs) and serum soluble CD137^8^. CD137^+^ Tregs are more suppressive than the CD137^-^ subset *in vitro* and are the primary cellular source of soluble CD137 among T cells^8^. Importantly, recombinant soluble CD137 prevents NOD mice from developing T1D^9^. These observations provide a possible mechanism of B10 *Idd9.3*-mediated T1D resistance and support *Tnfrsf9* as its underlying gene.

The purpose of this study was to develop a strategy that allows us to selectively express only the NOD or B10 *Tnfrsf9* allelic variant in an identical genetic background. With the recent advance in nuclease-based genetic engineering^10^, we directly targeted *Tnfrsf9* in both NOD and NOD.*Idd9*.*3*^*B10*^ mice and generated a pair of F1 hybrids mimicking coisogenic strains. We conclusively demonstrate that *Tnfrsf9* allelic difference directly controls the frequency of CD137^+^ Tregs and the level of serum soluble CD137. We also provide a general strategy for testing candidate genes in rodent genetic mapping studies.

## Results

### Generation of F1 hybrids that selectively express only NOD or B10 *Tnfrsf9*

**Figure 1:**
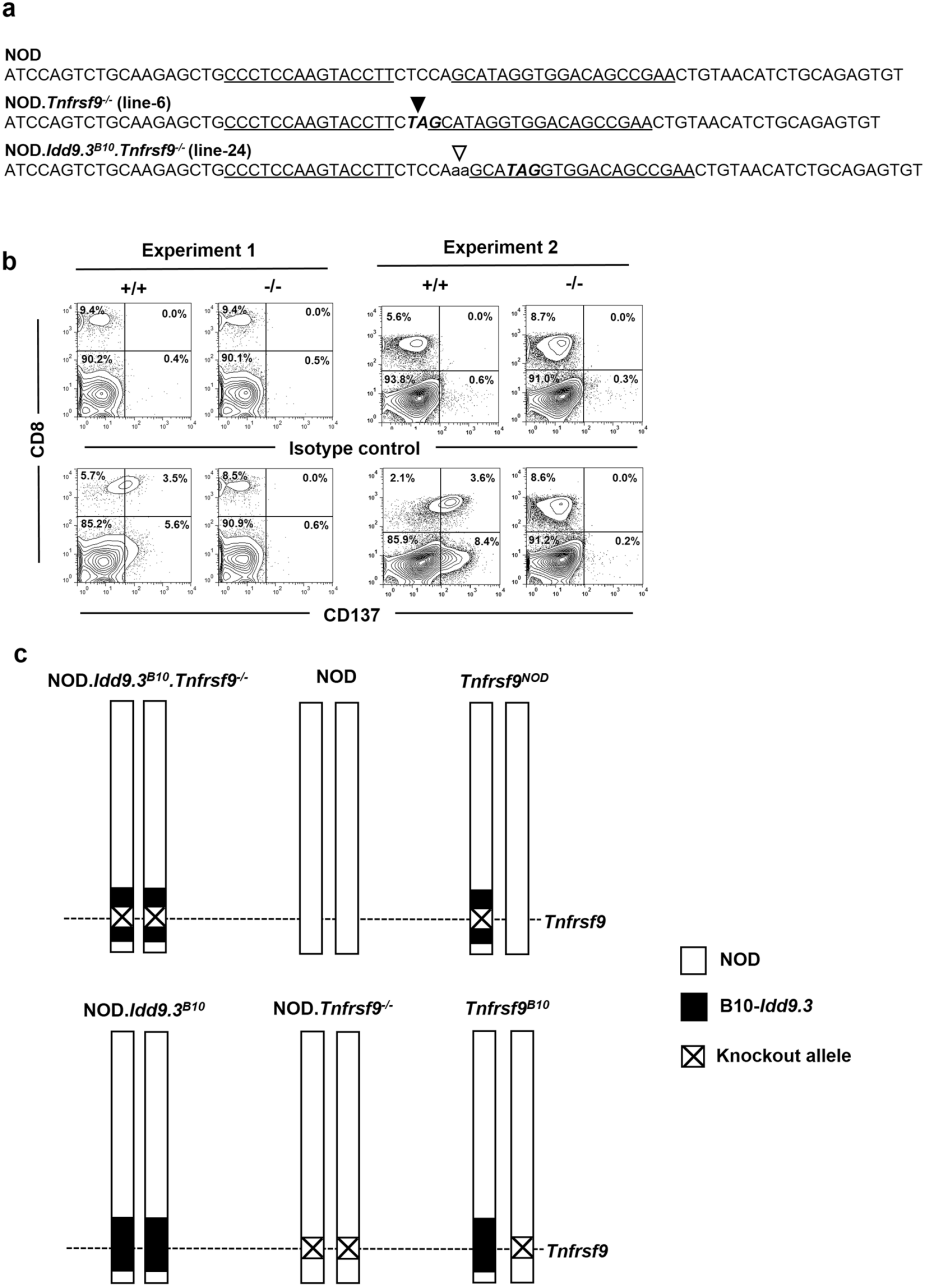
A general strategy for allele-specific expression of *Tnfrsf9* in genetically identical NOD background strains. (**a**) Comparing the mutant sequences of *Tnfrsf9* in our previously reported NOD.*Tnfrsf9*^−/−^ and the newly generated NOD.*Idd9.3*^*B10*^.*Tnfrsf9*^−/−^ strains to the wildtype NOD mice. A partial sequence of the *Tnfrsf9* second coding exon is shown. The wildtype sequence of the ZFN target site of the *Tnfrsf9* gene is shown above. The respectively altered sequences for NOD.*Tnfrsf9*^−/−^ and NOD.*Idd9.3*^*B10*^.*Tnfrsf9*^−/−^ are indicated below the wildtype. Each of the ZFN binding sequences on the opposite strands is underlined. The closed and open arrowheads respectively indicate the locations of the 2 base-pair deletion and 2 base-pair insertion (lower case) in the NOD.*Tnfrsf9*^−/−^ and NOD.*Idd9.3*^*B10*^.*Tnfrsf9*^−/−^ strains. The bold and italic letters depict the premature stop codons introduced as a result of the mutations at the ZFN target site. (**b**) Lack of CD137 protein expression in NOD.*Idd9.3*^*B10*^.*Tnfrsf9*^−/−^ mice confirms the knockout phenotype. Total splenocytes isolated from 15-week-old NOD.*Idd9.3*^*B10*^ (+/+) and NOD.*Idd9.3*^*B10*^.*Tnfrsf9*^−/−^ (−/−) male mice were stimulated with anti-CD3 (2.5 μg/ml) overnight and analyzed for CD137 expression by flow cytometry the following day. Cells were stained with anti-CD8 (for detecting CD8 T cells) and anti-CD137 or the corresponding isotype control antibody. The percentages of cells in the four quadrants of each plot are indicated. Results from two independent experiments are shown. (**c**) Schematic illustration of chromosome 4 in the parental NOD.*Idd9.3*^*B10*^.*Tnfrsf9*^−/−^, NOD, NOD.*Idd9.3*^*B10*^, and NOD.*Tnfrsf9*^−/−^ mice as well as the (NOD.*Idd9.3*^*B10*^.*Tnfrsf9*^−/−^ x NOD)F1 and (NOD.*Idd9*.3^*B10*^ x NOD.*Tnfrsf9*^−/−^)F1 mice (respectively indicated as *Tnfrsf9*^*NOD*^ and *Tnfrsf9*^*B10*^) that selectively express only the NOD or B10 *Tnfrsf9* gene. The B10-derived *Idd9.3* congenic region is not drawn to scale.

We previously targeted the second coding exon of the *Tnfrsf9* gene directly in NOD mice using a pair of zinc-finger nucleases (ZFNs) to generate the NOD.*Tnfrsf9*^−/−^ strain lacking CD137 expression^11^. Using the same ZFNs, we also successfully targeted *Tnfrsf9* directly in the NOD.*Idd9.3*^*B10*^ strain to create NOD.*Idd9.3*^*B10*^.*Tnfrsf9*^−/−^ mice. The mutations introduced in NOD.*Tnfrsf9*^−/−^ and NOD.*Idd9.3*^*B10*^.*Tnfrsf9*^−/−^ strains respectively deleted and inserted two base-pairs (Fig. 1a). While not identical, both mutations result in a premature stop codon at the target site (Fig. 1a). CD137 is upregulated on activated T cells, in particular CD8 T cells^12^. Thus, we stimulated total splenocytes isolated from NOD.*Idd9.3*^*B10*^ and NOD.*Idd9.3*^*B10*^.*Tnfrsf9*^−/−^ mice with anti-CD3 overnight to compare CD137 expression. As shown in Fig. 1b, CD137 protein was expressed on CD8 T cells isolated from NOD.*Idd9.3*^*B10*^ but not NOD.*Idd9.3*^*B10*^.*Tnfrsf9*^−/−^ mice, confirming the knockout phenotype. To determine the allelic effect of *Tnfrsf9*, we generated two types of F1 hybrids, (NOD.*Idd9.3*^*B10*^.*Tnfrsf9*^−/−^ x NOD)F1 and (NOD.*Idd9*.3^*B10*^ x NOD.*Tnfrsf9*^−/−^)F1, to respectively express NOD and B10 *Tnfrsf9* in the “identical” genetic background mimicking coisogenic strains (hereafter *Tnfrsf9*^*NOD*^ and *Tnfrsf9*^*B10*^ respectively) for subsequent analyses (Fig. 1c).

### Allelic difference in the *Tnfrsf9* gene controls the accumulation of CD137^+^ Tregs

**Figure 2:**
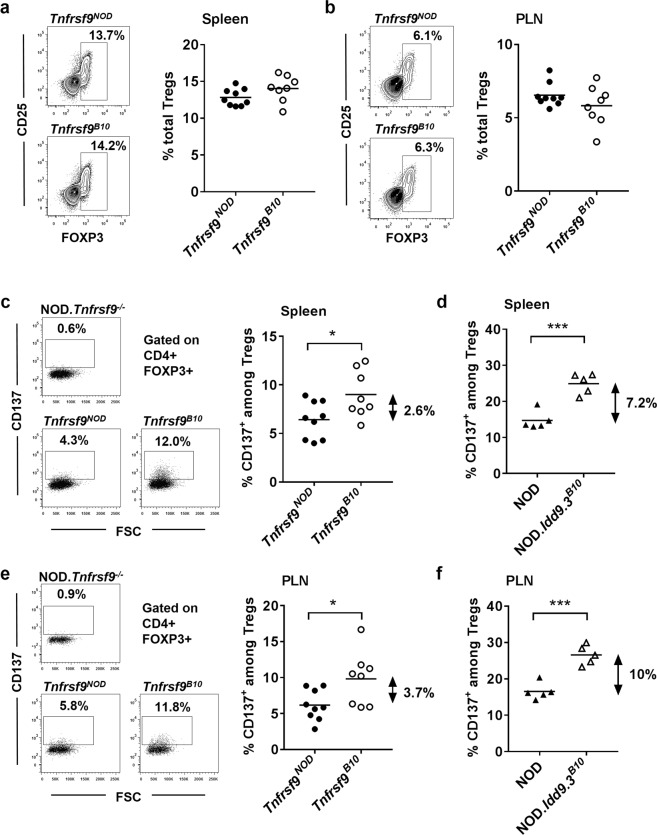
B10 *Tnfrsf9* increases the frequency of CD137^+^ Tregs. (**a**,**b**) The frequencies of total FOXP3^+^ Tregs are comparable in the spleen and PLN of *Tnfrsf9*^*NOD*^ and *Tnfrsf9*^*B10*^ F1 mice. (**a**) Representative flow cytometry profiles of CD25 and FOXP3 staining in splenic CD4 T cells of 9–12 week-old *Tnfrsf9*^*NOD*^ and *Tnfrsf9*^*B10*^ F1 female mice are shown on the left. Summarized results from three independent experiments are shown on the right. (**b**) Representative flow cytometry profiles of CD25 and FOXP3 staining in PLN CD4 T cells of 9–12 week-old *Tnfrsf9*^*NOD*^ and *Tnfrsf9*^*B10*^ F1 female mice are shown on the left. Summarized results from three independent experiments are shown on the right. (**c**) The percentage of CD137^+^ Tregs is increased in the spleen of *Tnfrsf9*^*B10*^ F1 mice. Representative flow cytometry profiles of CD137 staining in splenic Tregs of 9–11 week-old *Tnfrsf9*^*NOD*^ and *Tnfrsf9*^*B10*^ F1 female mice are shown on the left. Cells from a NOD.*Tnfrsf9*^−/−^ mouse was used as the negative control. Summarized results from three independent experiments are shown on the right. As indicated, the average frequency of CD137^+^ Tregs in the spleen of *Tnfrsf9*^*B10*^ F1 is 2.6% higher than that of *Tnfrsf9*^*NOD*^ F1 mice. *p < 0.05 by unpaired t test. (**d**) NOD.*Idd9.3*^*B10*^ mice have a higher frequency of splenic CD137^+^ Tregs than that of NOD mice. The results are summarized from 2 experiments using 9–12 week-old female mice. As indicated, the average frequency of splenic CD137^+^ Tregs in NOD.*Idd9.3*^*B10*^ is 7.2% higher than that of NOD mice. ***p < 0.0005 by unpaired t test. (**e**) The percentage of CD137^+^ Tregs is increased in the PLN of *Tnfrsf9*^*B10*^ F1 mice. Representative flow cytometry profiles of CD137 staining in PLN Tregs of 9–11 week-old *Tnfrsf9*^*NOD*^ and *Tnfrsf9*^*B10*^ F1 female mice are shown on the left. Cells from a NOD.*Tnfrsf9*^−/−^ mouse was used as the negative control. Summarized results from three independent experiments are shown on the right. As indicated, the average frequency of CD137^+^ Tregs in the PLN of *Tnfrsf9*^*B10*^ F1 is 3.7% higher than that of *Tnfrsf9*^*NOD*^ F1 mice. *p < 0.05 by unpaired t test. (**f**) NOD.*Idd9.3*^*B10*^ mice have a higher frequency of PLN CD137^+^ Tregs than that of NOD mice. The results are summarized from 2 independent experiments using 9–12 week-old female mice. As indicated, the average frequency of PLN CD137^+^ Tregs in NOD.*Idd9.3*^*B10*^ is 10% higher than that of NOD mice. ***p < 0.0005 by unpaired t test.

Except the origin of the functional *Tnfrsf9* allele, newly generated *Tnfrsf9*^*NOD*^ and *Tnfrsf9*^*B10*^ F1 mice are considered genetically identical. These F1 mice allowed us to determine if selective expression of NOD or B10 *Tnfrsf9* alone is sufficient to explain the phenotypic differences previously observed between NOD and NOD.*Idd9*.3^*B10*^ mice. T1D suppression in NOD.*Idd9*.3^*B10*^ mice is associated with a remarkably higher frequency of CD137^+^ Tregs^8^. Thus, we asked if this phenotype is controlled by the allelic difference between B10 and NOD *Tnfrsf9*. We first analyzed the frequencies of total Tregs and those that express CD137 in the spleen and pancreatic lymph node (PLN) of *Tnfrsf9*^*NOD*^ and *Tnfrsf9*^*B10*^ F1 mice. We observed similar percentages of total FOXP3^+^ Tregs in the spleen and PLN of *Tnfrsf9*^*NOD*^ and *Tnfrsf9*^*B10*^ F1 mice (Fig. 2a,b). Conversely, the frequency of CD137^+^ Tregs was higher in the spleen of *Tnfrsf9*^*B10*^ than *Tnfrsf9*^*NOD*^ F1 mice (Fig. 2c), although the difference between *Tnfrsf9*^*NOD*^ and *Tnfrsf9*^*B10*^ F1 hybrids was diminished relative to the comparison of NOD and NOD.*Idd9.3*^*B10*^ mice (Fig. 2d). This was likely because only one copy of functional *Tnfrsf9* was expressed in *Tnfrsf9*^*NOD*^ and *Tnfrsf9*^*B10*^ F1 mice. Similarly, the frequency of CD137^+^ Tregs was also increased in the PLN of *Tnfrsf9*^*B10*^ F1 progeny compared to that of *Tnfrsf9*^*NOD*^ F1 mice, but the difference was not as significant as what was observed between NOD and NOD.*Idd9.3*^*B10*^ mice (Fig. 2e,f).

### Allelic difference in the *Tnfrsf9* gene controls the level of serum soluble CD137

**Figure 3:**
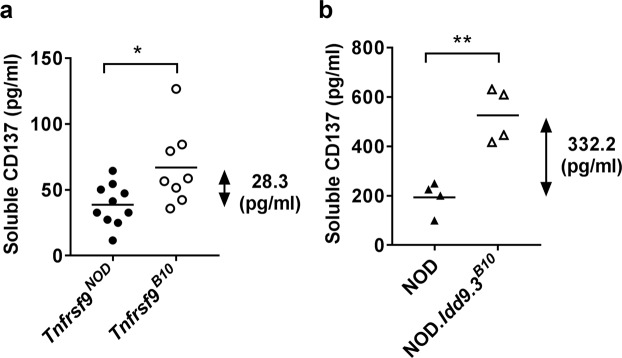
B10 *Tnfrsf9* increases the level of serum soluble CD137. (**a**) Serum soluble CD137 is higher in *Tnfrsf9*^*B10*^ than in *Tnfrsf9*^*NOD*^ F1 mice. Sera were collected from 10-week-old female mice and analyzed by ELISA for CD137. As indicated, the average level of serum soluble CD137 in *Tnfrsf9*^*B10*^ F1 is 28.3 pg/ml higher than that of *Tnfrsf9*^*NOD*^ F1 mice. *p < 0.05 by unpaired t test. (**b**) Serum soluble CD137 is higher in NOD.*Idd9.3*^*B10*^ than in NOD mice. Sera were collected from 10-week-old female mice and analyzed by ELISA for CD137. As indicated, the average level of serum soluble CD137 in NOD.*Idd9.3*^*B10*^ is 332.2 pg/ml higher than that of NOD mice. **p < 0.005 by unpaired t test.

CD137 is expressed as a membrane form or a soluble protein due to alternative splicing that removes the transmembrane domain^13^. Compared to NOD mice, the NOD.*Idd9*.3^*B10*^ congenic strain has a higher level of serum soluble CD137^8^. Thus, we asked if the level of serum soluble CD137 is also controlled by the allelic difference of *Tnfrsf9*. Sera were collected from *Tnfrsf9*^*NOD*^ and *Tnfrsf9*^*B10*^ F1 mice and analyzed for soluble CD137 by enzyme-linked immunosorbent assay (ELISA). We observed a significant increase of serum soluble CD137 in *Tnfrsf9*^*B10*^ compared to *Tnfrsf9*^*NOD*^ mice (Fig. 3a), albeit the difference was considerably reduced compared to that between NOD and NOD.*Idd9.3*^*B10*^ (Fig. 3b).

### T1D incidence in *Tnfrsf9*^*NOD*^ and *Tnfrsf9*^*B10*^ F1 mice

**Figure 4:**
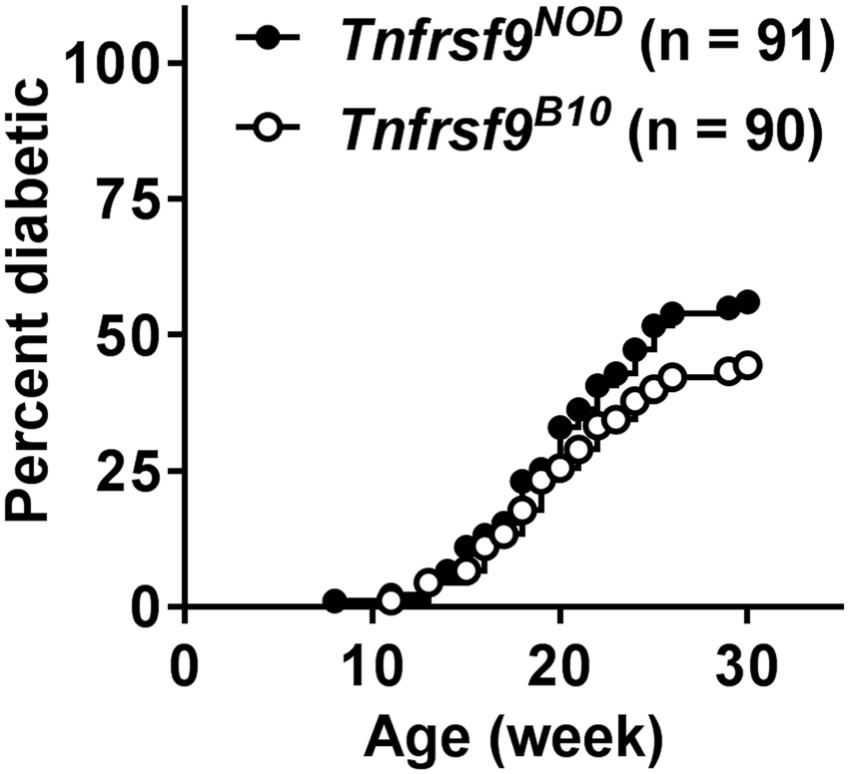
T1D incidence in *Tnfrsf9*^*NOD*^ and *Tnfrsf9*^*B10*^ F1 mice. *Tnfrsf9*^*B10*^ and *Tnfrsf9*^*NOD*^ F1 female mice were monitored for T1D development for 30 weeks. The incidence of T1D is not significantly different between *Tnfrsf9*^*B10*^ and *Tnfrsf9*^*NOD*^ F1 mice (p = 0.13 by Log-rank test).

Compared to NOD mice, T1D development is partially suppressed in the NOD.*Idd9.3*^*B10*^ congenic strain^5,7^. The *Tnfrsf9*^*NOD*^ and *Tnfrsf9*^*B10*^ F1 “coisogenic” strains provided an opportunity to directly test if *Tnfrsf9* variants are responsible for the T1D modulatory effect of the *Idd9.3* locus. To address this question, we monitored *Tnfrsf9*^*NOD*^ and *Tnfrsf9*^*B10*^ F1 females for T1D development. While there was a trend of reduced T1D incidence in *Tnfrsf9*^*B10*^ compared to *Tnfrsf9*^*NOD*^ F1 mice (p = 0.13 by Log-rank test), the difference did not reach statistical significance (Fig. 4). This could be due to the overall reduction of CD137 expression from a single *Tnfrsf9* copy in *Tnfrsf9*^*B10*^ and *Tnfrsf9*^*NOD*^ F1 mice that minimized the allele-specific functional differences important for regulating T1D development. This possibility is supported by the comparative analyses for the frequency of CD137^+^ Tregs and the level of serum soluble CD137 between *Tnfrsf9*^*B10*^ and *Tnfrsf9*^*NOD*^ F1 hybrids and between NOD and NOD.*Idd9.3*^*B10*^ mice as shown in Figs 2 and 3.

## Discussion

In this study we combined the power of congenic mapping and nuclease-based mutagenesis to establish a genetic strategy to directly compare two different alleles of the gene of interest in genetically identical strains. We tested this approach using the *Idd9.3* candidate gene *Tnfrsf9* in the NOD mouse model of T1D. NOD *Tnfrsf9* encodes a hypofunctional CD137 compared to the B10 variant^6^. The functional difference has been associated with an increased frequency of CD137^+^ Tregs in the NOD.*Idd9.3*^*B10*^ strain compared to standard NOD mice^8^. In the present study, we also detected a significant increase in the frequency of CD137^+^ Tregs in the spleen and PLN of our F1 mice expressing the B10 allele of *Tnfrsf9*. These results indicate that a more functional CD137 molecule alone leads to the accumulation of CD137^+^ Tregs, independent of other genes within the *Idd9.3* region or the differential state of diabetes progression in NOD and NOD.*Idd9.3*^*B10*^ mice. CD137^+^ Tregs exhibit superior function than their negative counterpart, possibly due to the production of soluble CD137^8^. Consistent with the previous study^8^, we also found a higher level of serum soluble CD137 in NOD.*Idd9.3*^*B10*^ than in NOD mice. We further show that a higher concentration of soluble CD137 was found in the serum of F1 mice expressing the B10 *Tnfrsf9* allele compared to those possessing the NOD allele. These results indicate that the *Tnfrsf9* allelic difference also directly controls the level of circulating soluble CD137. It is currently not known how alternative splicing of *Tnfrsf9* is regulated to give rise to membrane and soluble forms of CD137. Whether an increased level of serum soluble CD137 in mice carrying the B10 *Tnfrsf9* allele is a direct consequence of elevated CD137^+^ Tregs or involves regulation by other non-coding regions remains to be determined.

*Tnfrsf9* has been implicated as an *Idd9.3* underlying gene^6^. The *Tnfrsf9*^*NOD*^ and *Tnfrsf9*^*B10*^ F1 “coisogenic” strains allowed us to directly test if allelic difference in *Tnfrsf9* alone is sufficient to modulate T1D progression. Different from the results of the frequency of CD137^+^ Tregs and the level of serum soluble CD137, we did not observe significant T1D suppression in *Tnfrsf9*^*B10*^ compared to *Tnfrsf9*^*NOD*^ F1 mice. The lack of diabetes inhibition conferred by the B10 *Tnfrsf9* allele in our F1 mice could be that the functional difference conferred by one copy of B10 and NOD *Tnfrsf9* is not sufficient to impact T1D development. This possibility is supported by the diminished differences in the frequency of CD137^+^ Tregs and the level of serum soluble CD137 between *Tnfrsf9*^*NOD*^ and *Tnfrsf9*^*B10*^ F1 hybrids relative to the comparison between NOD and NOD.*Idd9.3*^*B10*^ mice (Figs 2 and 3). Another non-mutually exclusive explanation to the lack of T1D inhibition in *Tnfrsf9*^*B10*^ F1 mice is that other polymorphic gene(s) in the *Idd9.3* region also impact T1D development. One possible candidate is the non-coding micro RNA *Mir34a*. In a recent study it was found that the NOD.*Idd9.3* congenic strain had a significantly lower number of B cells than that in NOD mice^14^. The reduction of B cells in NOD.*Idd9.3* congenic strain correlated with a higher level of *Mir34a* that was thought to impair normal B cell development by negatively regulating FOXP1 expression^14^. However, we did not observe a reduction of B cells in the NOD.*Idd9.3*^*B10*^ congenic strain compared to NOD mice (Supplementary Fig. S1). The reason for the discrepancy is currently not known but could be due to differences in the housing environment. Immune cells are under the circadian control^15^. The circadian gene *Per3* represents another possible candidate in the *Idd9.3* region. A similar genetic approach reported here can be used to test additional genes within the *Idd9.3* region for their role in T1D development.

In conclusion, we demonstrate the feasibility to establish a “coisogenic” system by using a pair of F1 hybrids that selectively express an allele of the gene of interest for direct comparison of its functional consequence. The limitation of our approach is that only one copy of the gene is expressed, and the phenotypic difference may be quantitatively reduced if there is a dose-dependent effect. This possibility needs to be considered when testing candidate genes using our approach. Nevertheless, our strategy has the potential to be broadly applied to other experimental models to facilitate genetic mapping studies.

## Materials and Methods

### Mouse strains

NOD/ShiLtDvs (hereafter NOD) and NOD.B10Sn-*Idd9*.3^C57BL/10SnJ^/1106MrkTacJ (hereafter NOD.*Idd9.3*^*B10*^) were obtained from The Jackson Laboratory and subsequently maintained at the Medical College of Wisconsin MCW). NOD.*Tnfrsf9*^−/−^ mice (line-6), currently maintained at the N4 generation, have been previously reported^11^. NOD.*Idd9.3*^*B10*^.*Tnfrsf9*^−/−^ mice were generated using the previously described *Tnfrsf9* specific zinc-finger nuclease pairs designed, assembled, and validated by Sigma-Aldrich^11^. The condition of microinjection and methods of founder screening and genotyping have been described in detail^11^. A founder (line-24) with a two base-pair insertion at the target site, resulting in a premature stop codon (Fig. 1a), was backcrossed to NOD.*Idd9.3*^*B10*^ for 4 generations followed by intercrossing to fix the mutation to homozygosity. All mouse experimental protocols were carried out in accordance with the MCW Institutional Animal Care and Use Committee guidelines and approved by the committee.

### Flow cytometry analysis

Fluorochrome-labeled antibodies specific for CD8 (53-6.72), CD4 (RM4–5), CD3 (145-2C11), CD25 (7D4), CD137 (175B), Foxp3 (FJK-16s) were purchased from BD Biosciences (San Jose, CA), BioLegend (San Diego, CA), or eBioscience (San Diego, CA). Antibody staining and flow cytometry analysis procedures have been described previously^16^. For intracellular staining with FOXP3, samples were first stained for surface markers and then fixed/permeabilized for 2–4 hours using the FOXP3 staining buffer set from eBioscience. After fixation, the cells were washed twice with permeabilization buffer and then stained with anti-FOXP3 for 30 minutes. Stained cells were washed once with the FACS buffer and acquired using the FACSCalibur or LSRII flow cytometer (BD Biosciences). All flow cytometric data were analyzed with the FlowJo software (Tree Star, Ashland, OR). To analyze CD137 expression in NOD.*Idd9.3* and NOD.*Idd9.3.Tnfrsf9*^−/−^ mice, splenocytes from 15-week-old males were stimulated with 2.5 µg/ml anti-CD3 (145-2C11) overnight. Cells were then harvested and analyzed for CD137 protein expression by flow cytometry.

### Soluble CD137 enzyme-linked immunosorbent assay (ELISA)

Sera were collected from 10-week-old females of the indicated strains. The concentration of soluble CD137 was determined using the Mouse 4-1BB/TNFRSF9 DuoSet ELISA kit (R&D Systems). Soluble CD137 was not detectable in serum from NOD.*Tnfrsf9*^−/−^ or NOD.*Idd9.3*^*B10*^.*Tnfrsf9*^−/−^ mice, validating the specificity of the ELISA kit.

### Assessment of T1D

T1D development was monitored weekly using urine glucose strips (Diastix, Bayer) with onset defined by two consecutive readings of >250 mg/dl.

### Statistical analysis

Unpaired t test was used for comparison between two groups. Log-rank test was used for the analysis of T1D incidence. All statistical analyses were performed using the GraphPad Prism 6 software (La Jolla, CA).

## Supplementary information

Supplementary Figure S1
