# Supplementary material for: Combined congenic mapping and nuclease-based gene targeting for studying allele-specific effects of Tnfrsf9 within the Idd9.3 autoimmune diabetes locus
Source: Sci Rep. 2019 Mar 13;9:4316. doi: 10.1038/s41598-019-40898-8 (PMC6416332; doi:10.1038/s41598-019-40898-8)
Supplement: Supplementary file 1 — Supplementary Figure S1 [file 41598_2019_40898_MOESM1_ESM.pdf]

Combined congenic mapping and nuclease-based gene targeting for studying allele-specific effects of *Tnfrsf9* within the *Idd9.3* autoimmune diabetes locus

Matthew H. Forsberg, Bardees Foda, David V. Serreze, and Yi-Guang Chen

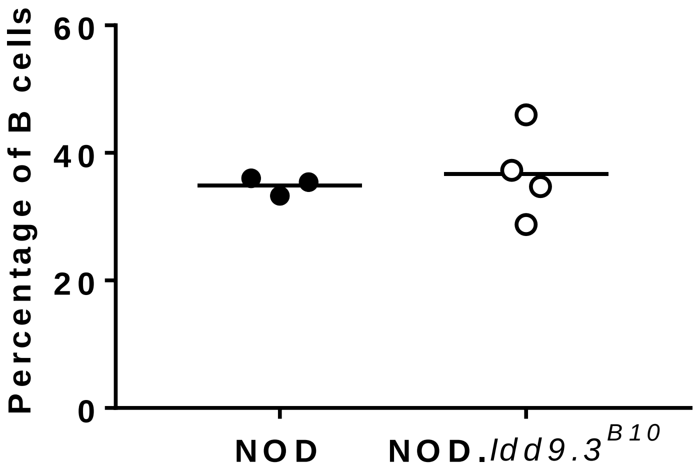

Supplementary Figure S1. The frequencies of splenic B cells in NOD and NOD.*Idd9.3*^{B10} mice are similar. The frequencies of B cells (B220+) were analyzed in the spleens of 7-8-week-old NOD and NOD.*Idd9.3*^{B10} mice by flow cytometry. The results from 2 experiments were combined.
